# Supplementary material for: Cultural transmission modes of music sampling traditions remain stable despite delocalization in the digital age
Source: PLoS One. 2019 Feb 5;14(2):e0211860. doi: 10.1371/journal.pone.0211860 (PMC6363214; doi:10.1371/journal.pone.0211860)
Supplement: S1 Appendix — (PDF) [file pone.0211860.s001.pdf]

Cultural transmission modes of music sampling traditions remain stable despite delocalization in the digital age

S1 Appendix

Mason Youngblood[a,b,1]

[a]Department of Psychology, The Graduate Center, City University of New York, New York, NY, USA
[b]Department of Biology, Queens College, City University of New York, Flushing, NY, USA
[1]myoungblood@gradcenter.cuny.edu

NBDA

0.1 Primary OADA

The results of the multiplicative NBDA model fit to the primary OADA with all four individual-level variables.

```
Summary of Multiplicative Social Transmission Model
Order of acquisition data
Unbounded parameterisation.

Coefficients:
                        Estimate    Bounded            se          z             p
Social transmission 1  1.334607e-01 0.1177462          NA         NA            NA
gender                -1.050416e-01        NA 4.017186e-02 -2.6148064 8.927804e-03
popularity            -1.335417e-02        NA 1.465003e-03 -9.1154550 0.000000e+00
followers             -9.611204e-08        NA 1.956053e-08 -4.9135695 8.943303e-07
meandist              -1.880816e-09        NA 1.361273e-08 -0.1381659 8.901093e-01


Likelihood Ratio Test for Social Transmission:

Null model includes all other specified variables
Social transmission and asocial learning assumed to combine multiplicatively

                            Df LogLik   AIC  AICc     LR p
With Social Transmission     5 7354.4 14719 14719 143.09 0
Without Social Transmission  4 7425.9 14860 14860
```

The results of all NBDA models fit to the primary OADA. In the "Additive?" column TRUE means the model was additive, FALSE means the model was multiplicative, and NA means the model was asocial. In the "ILVs", or individual-level variables, column the numbers correspond to the variables included in the model (1: gender; 2: popularity; 3: followers; 4: mean distance).

Additive?	ILVs	Social?	AICc	deltaAICc
FALSE	1 2 3 4	social	14718.7305939155	0
FALSE	2 3 4	social	14723.2581626229	4.53
FALSE	1 2 4	social	14749.7210576547	30.99
FALSE	2 4	social	14755.0075144196	36.28
FALSE	1 3 4	social	14795.6248094134	76.89
FALSE	3 4	social	14796.5052668663	77.77
FALSE	4	social	15956.6213244064	1237.89
FALSE	1 4	social	15957.1346280933	1238.4
FALSE	1 2 3	social	32229.4897515171	17510.76
FALSE	1 2	social	32260.0747802103	17541.34
FALSE	2 3	social	32267.3112186711	17548.58
FALSE	2	social	32298.2544538564	17579.52
FALSE	1 3	social	32356.8941850595	17638.16
FALSE	3	social	32389.9528866174	17671.22
FALSE	1	social	43609.4787479712	28890.75
TRUE	1	social	43612.45988987	28893.73
NA	0	social	43662.7306996825	28944
NA	1	asocial	43779.4569488198	29060.73
NA	0	asocial	43815.27922266	29096.55

```
TRUE        2        social   Inf              Inf
NA          2        asocial  Inf              Inf
TRUE        1 2      social   Inf              Inf
NA          1 2      asocial  Inf              Inf
TRUE        3        social   Inf              Inf
NA          3        asocial  Inf              Inf
TRUE        1 3      social   Inf              Inf
NA          1 3      asocial  Inf              Inf
TRUE        2 3      social   Inf              Inf
NA          2 3      asocial  Inf              Inf
TRUE        1 2 3    social   Inf              Inf
NA          1 2 3    asocial  Inf              Inf
TRUE        4        social   Inf              Inf
NA          4        asocial  Inf              Inf
TRUE        1 4      social   Inf              Inf
NA          1 4      asocial  Inf              Inf
TRUE        2 4      social   Inf              Inf
NA          2 4      asocial  Inf              Inf
TRUE        1 2 4    social   Inf              Inf
NA          1 2 4    asocial  Inf              Inf
TRUE        3 4      social   Inf              Inf
NA          3 4      asocial  Inf              Inf
TRUE        1 3 4    social   Inf              Inf
NA          1 3 4    asocial  Inf              Inf
TRUE        2 3 4    social   Inf              Inf
NA          2 3 4    asocial  Inf              Inf
TRUE        1 2 3 4  social   Inf              Inf
NA          1 2 3 4  asocial  Inf              Inf
```

0.2 Additional OADA

The eight songs in the "Most Sampled Tracks" on WhoSampled that were released after 1990. The fifth song, "I'm Good" by YG, was excluded from the additional OADA because it is a producer tag used by a single artist.

1. "Crash Goes Love (Yell Apella)" by Loleatta Holloway (1992)
2. "Shook Ones Part II" by Mobb Deep (1994)
3. "C.R.E.A.M." by Wu-Tang Clan (1993)
4. "Sound of Da Police" by KRS-One (1993)
5. "I'm Good" by YG (2011) [excluded producer tag]
6. "Juicy" by The Notorious B.I.G. (1994)
7. "Sniper" by DJ Trace and Pete Parsons (1999)
8. "Who U Wit?" by Lil Jon and The East Side Boyz (1997)

The results of the additive NBDA model fit to the additional OADA. Remember that the fifth song was excluded, so the transmission estimates for five, six, and seven here are actually for six, seven, and eight.

```
Summary of Additive Social Transmission Model
Order of acquisition data
Unbounded parameterisation

Coefficients
                         Estimate    Bounded
Social transmission 1 0.13558602 0.11939740
Social transmission 2 0.28805974 0.22363849
Social transmission 3 0.05184340 0.04928814
Social transmission 4 0.60154771 0.37560399
Social transmission 5 0.06816578 0.06381573
Social transmission 6 0.07600555 0.07063677
Social transmission 7 0.01547410 0.01523830


Likelihood Ratio Test for Social Transmission:

Null model includes all other specified variables
Social transmission and asocial learning assumed to combine additively

                            Df LogLik   AIC  AICc     LR  p
With Social Transmission     7   6450 12914 12914 101.99  0
Without Social Transmission  0   6501 13002 13002
```

STERGM

The results of all formation models of the STERGM fit to the data from 1984-1999. In the "ILVs", or individual-level variables, column the numbers correspond to the variables included in the model (1: gender; 2: popularity; 3: followers; 4: mean distance).

```
    ILVs       AIC  deltaAIC
   1 2 4  7676.380   0.00000
 1 2 3 4  7678.174   1.79348
     1 2  7693.425  17.04473
   1 2 3  7695.203  18.82240
     1 4  7702.003  25.62300
   1 3 4  7702.160  25.77921
     2 4  7710.403  34.02256
   2 3 4  7711.909  35.52898
       1  7718.147  41.76695
     1 3  7718.390  42.00953
       2  7727.194  50.81323
     2 3  7728.701  52.32017
       4  7737.373  60.99249
     3 4  7738.094  61.71319
       0  7753.228  76.84742
       3  7753.985  77.60457
```

The results of the best-fitting formation model of the STERGM with the most individual-level variables fit to the data from 1984-1999.

```
==========================
Summary of model fit
==========================

Formula:   y.form ~ edges + nodecov("meandist") + absdiff("popularity") +
    absdiff("followers") + nodematch("gender", diff = TRUE)
<environment: 0x1cb31da8>

Iterations:  11 out of 20

Monte Carlo MLE Results:
                      Estimate Std. Error MCMC % z value Pr(>|z|)
edges               -9.382e+00  1.089e-01      0 -86.175  < 1e-04 ***
nodecov.meandist    -1.292e-07  3.371e-08      0  -3.833 0.000126 ***
absdiff.popularity  -1.618e-02  3.284e-03      0  -4.927  < 1e-04 ***
absdiff.followers    1.194e-08  2.579e-08      0   0.463 0.643396
nodematch.gender.-1  1.926e+00  3.293e-01      0   5.851  < 1e-04 ***
nodematch.gender.0   3.478e-01  1.965e-01      0   1.770 0.076773 .
nodematch.gender.1   5.313e-01  1.117e-01      0   4.757  < 1e-04 ***
---
Signif. codes:  0 '***' 0.001 '**' 0.01 '*' 0.05 '.' 0.1 ' ' 1

     Null Deviance: 6349494  on 4580192  degrees of freedom
 Residual Deviance:    7664  on 4580185  degrees of freedom

AIC: 7678    BIC: 7772    (Smaller is better.)
```

The results of the goodness-of-fit analysis of the formation model of the STERGM with the most individual-level variables fit to the data from 1984-1999.

```
Goodness-of-fit for degree

     obs   min     mean   max MC p-value
0  10698 10388 10484.50 10553       0.00
1    801  1032  1101.97  1200       0.00
2    148    89   104.41   126       0.00
3     43    16    20.71    29       0.00
4     15     5     8.01    11       0.00
5      0     0     0.38     2       1.00
6      6     0     0.95     2       0.00
7      1     0     0.07     1       0.14
8     12     4     6.55     7       0.00
9      1     0     0.42     3       0.62
10     1     0     0.03     1       0.06
11     2     0     0.91     1       0.00
12     2     0     1.00     2       0.14
13     0     0     0.08     1       1.00
14     0     0     0.01     1       1.00

Goodness-of-fit for edgewise shared partner

     obs min   mean max MC p-value
```

```
esp0  533  608  645.34  700          0
esp1   92   43   43.01   44          0
esp2   42   19   19.01   20          0
esp3    6    3    3.00    3          0
esp7   71   36   36.00   36          0
esp8    1    0    0.00    0          0

Goodness-of-fit for minimum geodesic distance

         obs      min        mean      max MC p-value
1        745      709      746.36      801       0.92
2        421      186      212.11      249       0.00
3        243       46       67.21      102       0.00
4        138       14       22.30       45       0.00
5         60        6        8.67       22       0.00
6         19        0        1.07        7       0.00
7          5        0        0.06        3       0.00
Inf 68788954 68789399 68789527.22 68789605       0.00

Goodness-of-fit for model statistics

                                 obs           min          mean           max MC p-value
edges                         745.00        709.00        746.36        801.00       0.92
nodecov.meandist        -298388294.47 -366967354.28 -300622250.67 -216526380.89       0.94
absdiff.popularity          16092.87      15023.48      16140.46      17764.91       0.94
absdiff.followers       631702714.34  538012952.78  636527414.86  770413121.94       1.00
nodematch.gender.-1            20.00         13.00         20.16         28.00       1.00
nodematch.gender.0             68.00         56.00         68.37         80.00       1.00
nodematch.gender.1            384.00        361.00        385.54        424.00       1.00
```

The results of all formation models of the STERGM fit to the data from 2000-2017. In the "ILVs", or individual-level variables, column the numbers correspond to the variables included in the model (1: gender; 2: popularity; 3: followers; 4: mean distance).

```
   ILVs      AIC  deltaAIC
  1 2 3 14194.16   0.00000
1 2 3 4 14196.11   1.94688
    2 3 14365.84 171.67787
  2 3 4 14367.30 173.13943
    1 2 14409.22 215.06168
  1 2 4 14410.98 216.82145
    1 3 14563.71 369.55351
  1 3 4 14565.71 371.55212
      2 14599.55 405.39275
    2 4 14600.59 406.43284
      1 14636.97 442.80822
    1 4 14638.95 444.78763
      3 14748.97 554.80763
    3 4 14750.71 556.55346
      0 14836.01 641.84666
      4 14837.57 643.40921
```

The results of the best-fitting formation model of the STERGM with the most individual-level variables fit to the data from 2000-2017.

```
==========================
Summary of model fit
==========================

Formula:   y.form ~ edges + nodecov("meandist") + absdiff("popularity") +
    absdiff("followers") + nodematch("gender", diff = TRUE)
<environment: 0x1ad107308>

Iterations:  10 out of 20

Monte Carlo MLE Results:
                      Estimate Std. Error MCMC %  z value Pr(>|z|)
edges               -8.530e+00  7.639e-02      0 -111.660  < 1e-04 ***
nodecov.meandist    -3.907e-09  1.702e-08      0   -0.230  0.81847
absdiff.popularity  -4.764e-02  2.799e-03      0  -17.022  < 1e-04 ***
absdiff.followers    1.726e-07  9.188e-09      0   18.786  < 1e-04 ***
nodematch.gender.-1  8.030e-01  3.605e-01      0    2.227  0.02592 *
nodematch.gender.0  -7.953e-01  2.582e-01      0   -3.080  0.00207 **
nodematch.gender.1   8.877e-01  7.878e-02      0   11.269  < 1e-04 ***
---
Signif. codes:  0 '***' 0.001 '**' 0.01 '*' 0.05 '.' 0.1 ' ' 1

     Null Deviance: 7195553  on 5190494  degrees of freedom
 Residual Deviance:   14182  on 5190487  degrees of freedom

AIC: 14196    BIC: 14290    (Smaller is better.)
```

The results of the goodness-of-fit analysis of the formation model of the STERGM with the most individual-level variables fit to the data from 2000-2017 are below.

Goodness−of−fit for degree

	obs	min	mean	max	MC p−value
0	11179	10679	10811.81	10930	0.00
1	1507	1878	2005.54	2115	0.00
2	371	313	351.05	398	0.24
3	130	76	88.47	104	0.00
4	68	17	25.10	31	0.00
5	26	3	7.85	13	0.00
6	5	0	1.94	6	0.06
7	5	0	1.94	3	0.00
8	0	0	0.26	2	1.00
9	2	0	0.04	1	0.00
10	1	0	0.00	0	0.00

Goodness−of−fit for edgewise shared partner

	obs	min	mean	max	MC p−value
esp0	1176	1324	1379.71	1458	0
esp1	294	144	144.42	147	0
esp2	75	35	36.03	37	0
esp3	22	10	10.02	11	0

Goodness−of−fit for minimum geodesic distance

	obs	min	mean	max	MC p−value
1	1567	1514	1570.18	1648	0.90
2	1189	607	677.37	735	0.00
3	1107	320	373.55	436	0.00
4	950	177	225.67	286	0.00
5	658	83	123.00	177	0.00
6	380	37	69.22	125	0.00
7	182	15	39.00	82	0.00
8	79	3	19.09	47	0.00
9	16	1	8.26	34	0.22
10	1	0	2.59	14	1.00
11	0	0	0.58	7	1.00
12	0	0	0.12	4	1.00
13	0	0	0.02	1	1.00
Inf	88352442	88355141	88355462.35	88355763	0.00

Goodness−of−fit for model statistics

	obs	min	mean	max	MC p−value
edges	1567.00	1514.00	1570.18	1.648000e+03	0.90
nodecov.meandist	−52499302.28	−196776194.24	−55329463.86	1.010890e+08	0.96
absdiff.popularity	25911.58	24355.87	25912.42	2.714093e+04	0.98
absdiff.followers	2958736083.69	2685506866.45	2938426320.54	3.215706e+09	0.86
nodematch.gender.−1	16.00	10.00	16.04	2.400000e+01	1.00
nodematch.gender.0	31.00	23.00	31.33	4.200000e+01	1.00
nodematch.gender.1	1012.00	956.00	1014.21	1.069000e+03	0.94

The results of the formation models of the STERGM with all individual-level variables assuming different transition years. The top and bottom tables show the results for before and after each transition year. The number of unique artists in each time period is included in the second row. Regardless of the transition year, mean distance and gender (F and M) had the same significance pattern and direction of effect observed in the main analysis. The results for popularity only varied from the main analysis in the first time period when the transition year was 1994 or 1996, which could be the result of lower sample sizes. The results for followers were consistent with the main analysis in the second time period, but fluctuated dramatically across transition years in the first time period.

	Pre−1994		Pre−1996		Pre−1998		Pre−2000	
	n = 205		n = 286		n = 370		n = 450	
	Estimate	p−value	Estimate	p−value	Estimate	p−value	Estimate	p−value
Mean Distance	−2.0e−07	9.0e−04	−1.0e−07	1.1e−02	−1.1e−07	3.2e−03	−1.3e−07	1.3e−04
Popularity	−5.1e−03	2.9e−01	−5.9e−03	1.6e−01	−1.2e−02	1.5e−03	−1.6e−02	8.4e−07
Followers	−2.3e−07	8.0e−03	−2.4e−07	1.9e−03	−8.3e−08	5.5e−02	1.2e−08	6.4e−01
Gender (F)	1.5e+00	1.0e−02	1.3e+00	3.1e−02	1.3e+00	1.3e−02	1.9e+00	4.9e−09
Gender (M)	5.8e−02	7.3e−01	2.6e−01	7.4e−02	4.7e−01	1.4e−04	5.3e−01	2.0e−06

Pre−2002		Pre−2004		Pre−2006	
n = 520		n = 593		n = 659	
Estimate	p−value	Estimate	p−value	Estimate	p−value
−1.1e−07	1.6e−04	−1.2e−07	1.2e−05	−1.1e−07	2.0e−05
−1.8e−02	1.7e−09	−2.0e−02	9.9e−13	−2.2e−02	4.4e−17
2.8e−08	2.0e−01	3.9e−08	4.3e−02	4.6e−08	8.0e−03
1.8e+00	5.1e−09	1.7e+00	6.1e−08	1.5e+00	1.6e−06
5.7e−01	2.0e−08	6.7e−01	5.8e−13	6.7e−01	7.5e−15

	Post−1994		Post−1996		Post−1998		Post−2000	
	n = 876		n = 836		n = 781		n = 725	
	Estimate	p−value	Estimate	p−value	Estimate	p−value	Estimate	p−value
Mean Distance	−2.0e−08	2.1e−01	−2.2e−08	1.7e−01	−9.3e−09	5.8e−01	−3.9e−09	8.2e−01
Popularity	−4.3e−02	9.3e−72	−4.4e−02	2.4e−70	−4.7e−02	1.7e−68	−4.8e−02	5.7e−65
Followers	1.6e−07	2.6e−74	1.6e−07	1.6e−77	1.7e−07	1.1e−75	1.7e−07	9.9e−79
Gender (F)	1.3e+00	3.7e−07	1.3e+00	9.2e−07	8.3e−01	1.4e−02	8.0e−01	2.6e−02
Gender (M)	8.9e−01	7.3e−37	9.0e−01	2.0e−35	8.6e−01	2.8e−30	8.9e−01	1.9e−29

	Post−2002		Post−2004		Post−2006	
	n = 667		n = 607		n = 544	
	Estimate	p−value	Estimate	p−value	Estimate	p−value
	1.2e−09	9.4e−01	1.7e−08	3.4e−01	2.2e−08	2.6e−01
	−4.9e−02	3.4e−61	−5.3e−02	2.4e−58	−5.5e−02	3.1e−52
	1.8e−07	3.2e−77	1.9e−07	1.9e−81	2.0e−07	1.2e−83
	8.7e−01	1.5e−02	9.7e−01	7.6e−03	1.2e+00	1.4e−03
	8.6e−01	1.5e−25	8.4e−01	4.7e−21	9.2e−01	5.6e−21
