# Supplementary figures and images for: Cultural transmission modes of music sampling traditions remain stable despite delocalization in the digital age
Source: PLoS One. 2019 Feb 5;14(2):e0211860. doi: 10.1371/journal.pone.0211860 (PMC6363214; doi:10.1371/journal.pone.0211860)

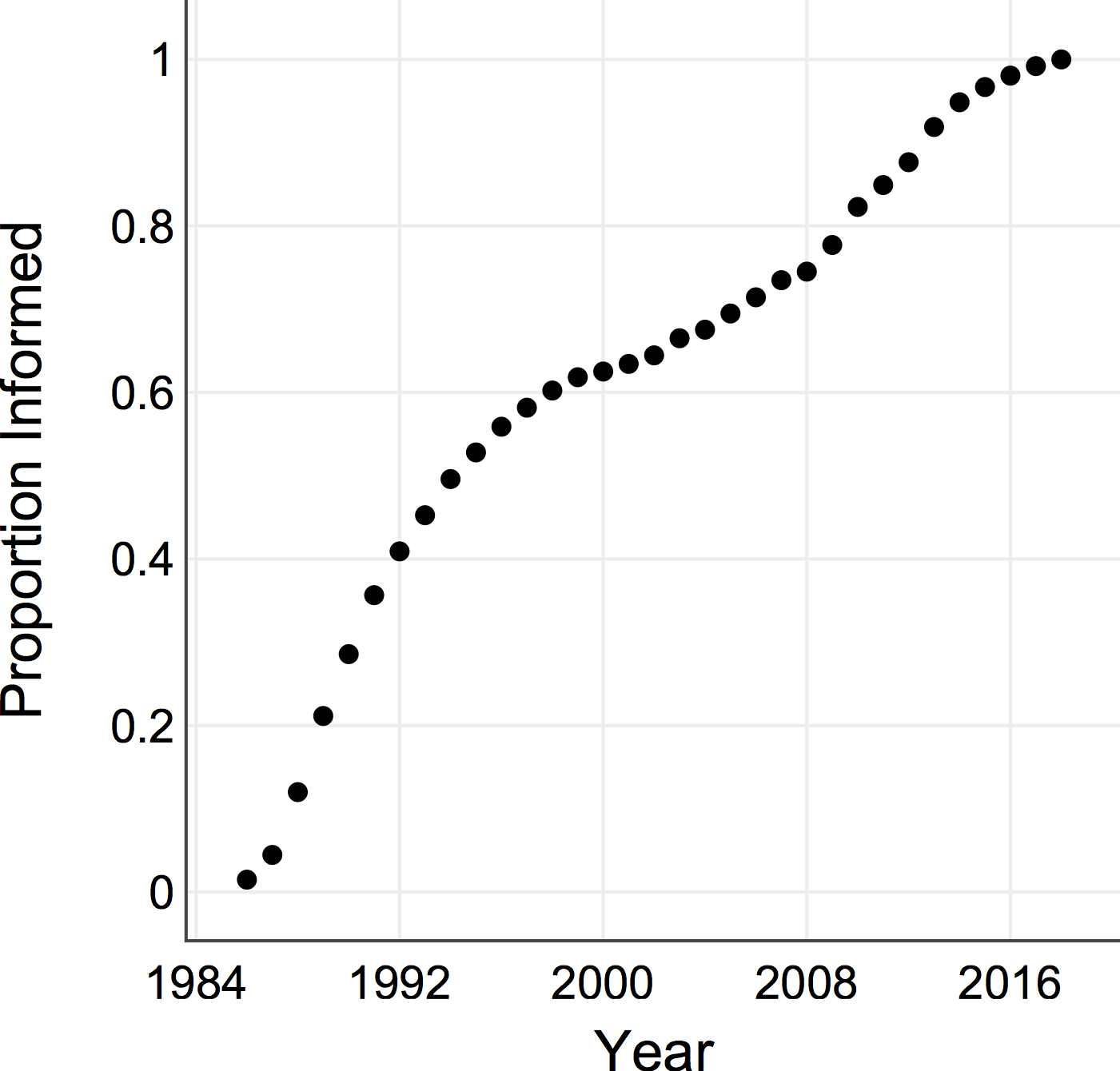

Supplement: S1 Fig — The proportion of informed individuals is on the y-axis, and the year is on the x-axis. Although recent research suggests that inferring acquisition modes from diffusion curves is unreliable, it appears that the curve may have the S-shape indicative of social transmission prior to the early-2000s. (TIFF) [file pone.0211860.s002.tiff]

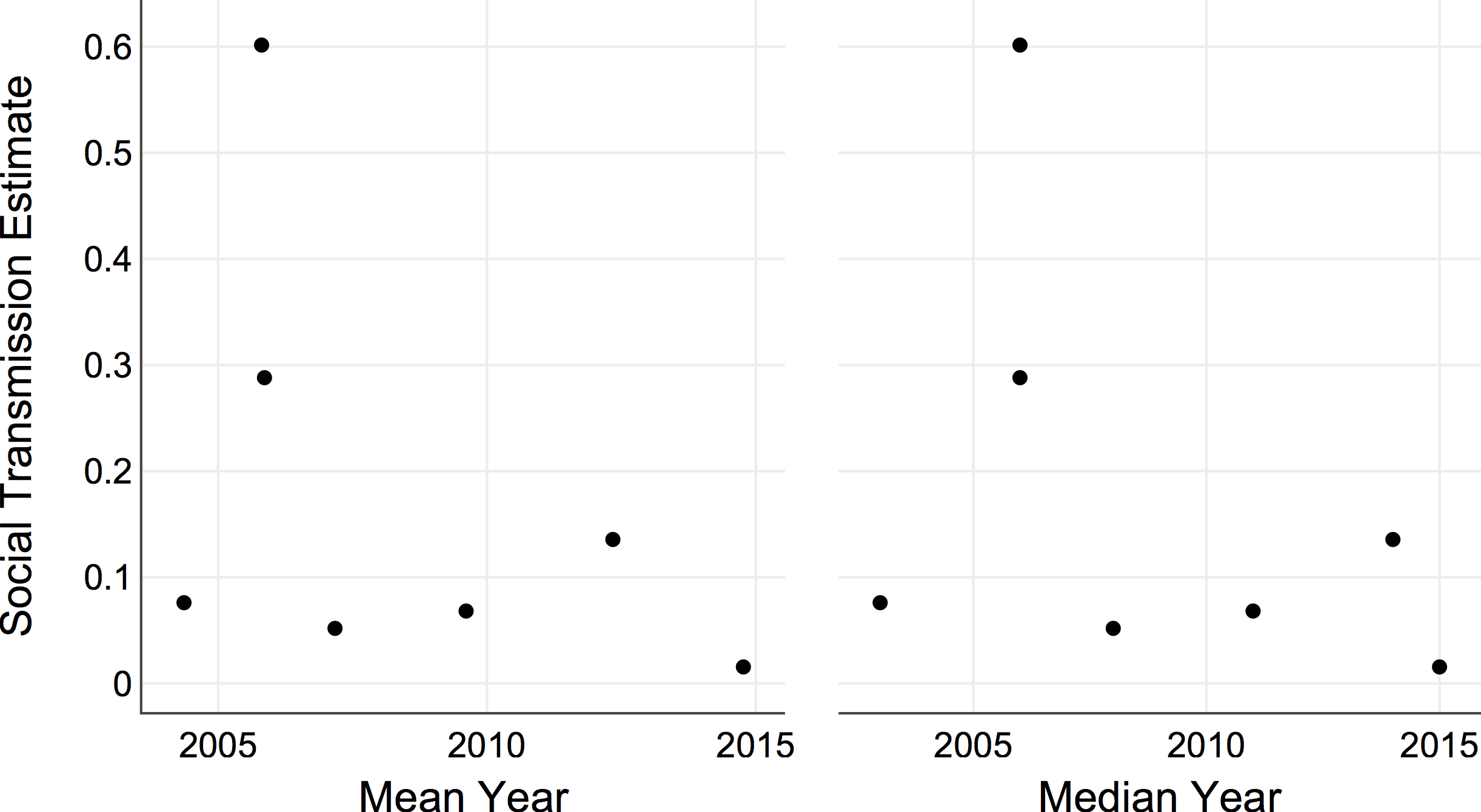

Supplement: S2 Fig — The mean (left) and median (right) years of diffusion are on the x-axis, and the social transmission estimates from the additive model are on the y-axis. Linear regression found no significant relationships between either mean year of diffusion and social transmission estimate (R2 = 0.20, p = 0.31) or median year of diffusion and social transmission estimate (R2 = 0.17, p = 0.36). (TIFF) [file pone.0211860.s003.tiff]

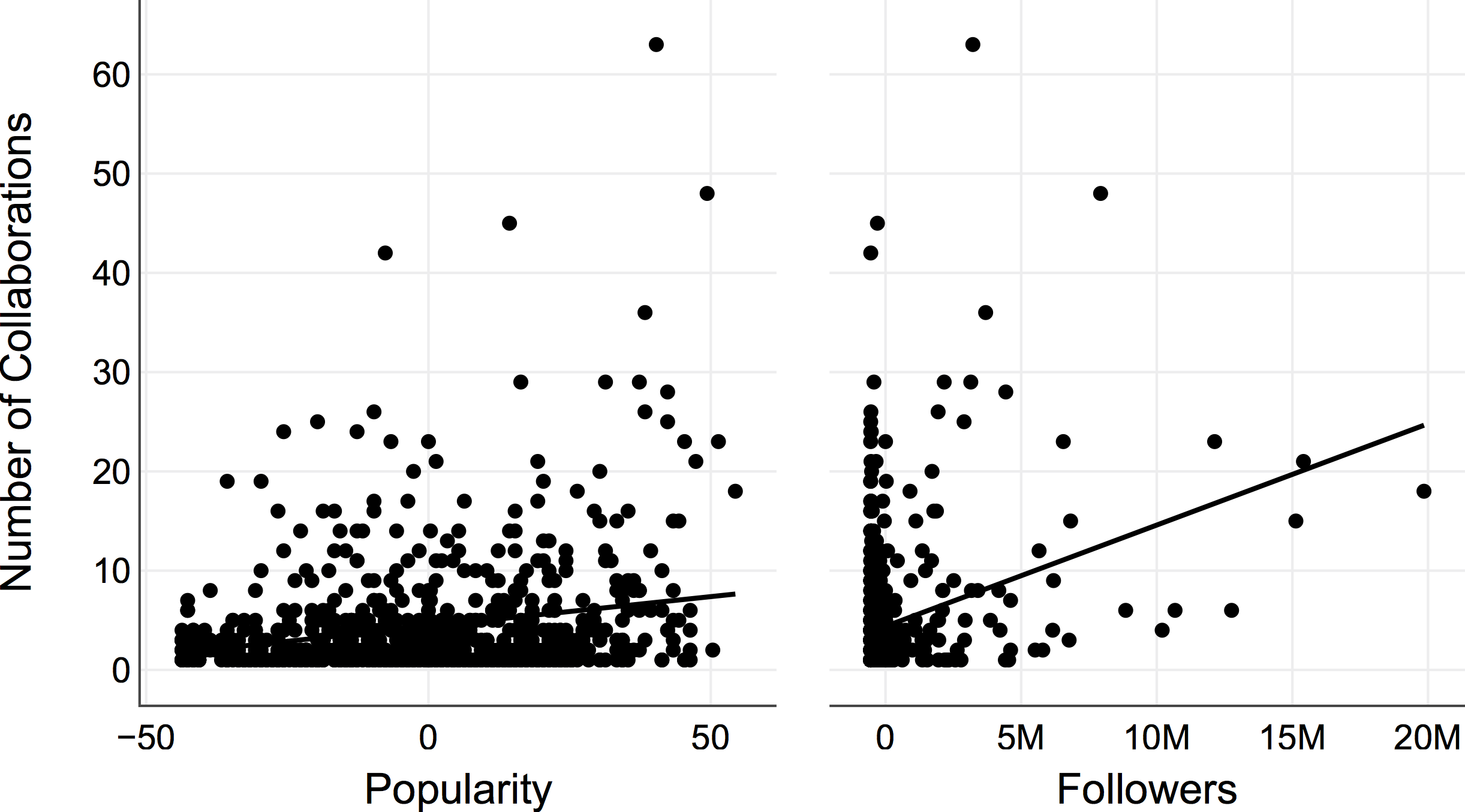

Supplement: S3 Fig — Popularity and followers are on the x-axis, and number of collaborations is on the y-axis. Linear regression found significant positive relationships between both popularity and number of collaborations (R2 = 0.048, p < 0.001) and followers and number of collaborations (R2 = 0.090, p < 0.001). (TIFF) [file pone.0211860.s004.tiff]
